# Study on AgInZnS-Graphene Oxide Non-toxic Quantum Dots for Biomedical Sensing

**DOI:** 10.3389/fchem.2020.00331

**Published:** 2020-05-05

**Authors:** Chi Song, Haoyue Luo, Xiaogang Lin, Zhijia Peng, Lingdong Weng, Xiaosheng Tang, Shibin Xu, Ming Song, Lifeng Jin, Xiaodong Zheng

**Affiliations:** ^1^Department of Life Science and Technology, Changshu Institute of Technology, Changshu, China; ^2^Key Laboratory of Optoelectronic Technology and Systems of Ministry of Education of China, Chongqing University, Chongqing, China; ^3^Chongqing University Cancer Hospital, Chongqing University, Chongqing, China

**Keywords:** quantum dots, AgInZnS-graphene oxide, non-toxic, interaction mechanism, human serum albumin, spectroscopy

## Abstract

In recent years, non-toxic quantum dot has caught the attention of biomedical fields. However, the inherent cytotoxicity of QDs makes its biomedical application painful, and is a major drawback of this method. In this paper, a non-toxic and water-soluble quantum dot AgInZnS-GO using graphene oxide was synthesized. A simple model of state complex was also established, which is produced through a combination of quantum dots and protein. The interaction between AIZS-GO QDs and human serum albumin (HSA) has significant meaning *in vivo* biological application. Herein, the binding of AIZS-GO QDs and HSA were researched using fluorescence spectra, Uv-visible absorption spectra, FT-IR spectra, and circular dichroism (CD) spectra. The results of fluorescence spectra demonstrate that AIZS-GO QDs have an obvious fluorescence quenching effect on HSA. The quenching mechanism is static quenching, which implies that some type of complex was produced by the binding of QDs and HSA. These results were further proved by Uv-visible absorption spectroscopy. The Stern-Volmer quenching constant K_sv_ at various temperatures (298 K, 303 K, 308 K) were acquired from analyzing Stern-Volmer plots of the fluorescence quenching information. The Van't Hoff equation could describe the thermodynamic parameters, which demonstrated that the van der Waals and hydrogen bonds had an essential effect on the interaction. FT-IR spectra and CD spectra further indicate that AIZS-GO QDs can alter the structure of HSA. These spectral methods show that the quantum dot can combine well with HSA. The experimental results showed that AgInZn-GO water-soluble quantum dots have good biocompatibility, which can be combined with proteins to form new compounds which have no cytotoxicity and biological practicability. It provides an important basis for the combination of quantum dots and specific proteins as well as fluorescent labeling.

## Introduction

In recent years, researchers have devoted themselves to the development of various nanomaterials and their manufacturing methods, which have opened up new research directions for nano-biotechnology in the field of biomedicine. Due to its special optical and physical properties, quantum dots (QDs) have caught the attention of various research fields (Alivisatos, 1996; Dabbousi et al., 1997; Niemeyer, 2001; Fu and Lakowicz, 2006; Jiang et al., 2018; Tang et al., 2019; Wang et al., 2019). Quantum dots can be used as fluorescent markers to detect proteins, DNA, and specific proteases qualitatively or quantitatively. In general, reactions will occur between QDs and proteins because of the diverse modified coating agents and elemental composition on the surface of QDs (Bruchez et al., 1998; Chan and Nie, 1998). However, the inherent cytotoxicity of QDs makes the biomedical application painful, a major drawbacks of the method. For instance, many studies reported the influence CdTe and CdSe quantum dots have on protein. The toxicity of Cd, Te, and Se elements remains a problem for practical application and makes its use non-negligible (Nirmal et al., 1994; Shiang et al., 1995; Ragab et al., 2014). In response to the above issues, some researches refer to the I-III-VI_2_ type QDs (Allen and Bawendi, 2008) like AgInS_2_ (Hamanaka et al., 2011), CuInS_2_ (Cassette et al., 2010; Li et al., 2015) and ZnS-AgInS_2_ (Torimoto et al., 2007).

In this paper, we focus on novelty AgInZnS (AIZS) non-toxic quantum dots. AIZS QDs are prevalent in diverse fields because of their optical and electrical characteristics, which include tunable-emission wavelength, high absorbance coefficient, and high quantum yield (Sheng et al., 2012). The as-prepared hydrophobic AIZS QDs should be converted into a hydrophilic polymer before reacting with the protein. Compared with other phase transfer agents, strong absorption ability was shown in graphene oxide (GO) (Gao et al., 2013; Shin et al., 2014; Farid et al., 2016). Due to its biocompatibility, non-toxicity, and excellent water solubility, GO is suitable to transfer the hydrophobic AIZS QDs in hydrophilic polymers and for further biological application (Zu et al., 2016).

HSA is broadly distributed in blood plasma. It has a significant effect on equilibrium osmotic pressure and transports various molecules in blood (Carter et al., 1989; Dong et al., 1990; Leckband, 2000; Lin et al., 2019a). Nanoparticles and drugs can bind with HSA for their exogenous ligand and is then transported into the body's circulatory system. Because there are some interactions between nanoparticles and the protein, it is necessary to analyze the influence of nanoparticles on HSA at the molecular level after it enters the human body. In many biophysical and biochemical studies, HSA has been diffusely utilized as a model protein because of its medicinal value and its unique ligand binding properties (Figge et al., 1991).

As far as we know, AIZS-GO QDs has enormous potential in biological research. At first, we wanted to establish a molecular model of interaction *in vitro* between quantum dots and proteins. In this paper, the specific biomedical research contents mainly include the following three aspects. First, the preparation of the water-soluble quantum dot AgInZnS-GO with high biocompatibility. The particle size and distribution of quantum dots were observed by transmission electron microscopy, and the carboxyl group was distributed according to the Fourier transform infrared spectroscopy. Second, the effects of the AgInZnS-GO quantum dot with HSA was studied using a multispectral technique. In this paper, the quenching mechanism of the quantum dot and protein interaction was analyzed by fluorescence spectrometry. The ultraviolet and visible absorption spectra is used to determine whether a new ground state complex was generated. The structural changes of protein and the major chemical bond changes during the reaction were analyzed by the Fourie transform infrared spectra and the circular dichromatic spectrum. Third, the cytotoxicity of quantum dots was analyzed. The safe concentration and time range of quantum dots were analyzed from two perspectives: cell appreciation rate and cell morphology. Next, we will perform a binding study *in vivo* in animal models.

## Materials and Methods

### Materials

HSA was purchased from Sigma-Aldrich (Sigma, St, Louis, MO, USA), and its purity was greater than 99.9%, containing very few fatty acids. HSA was dissolved in 0.058 M Tris-HCL buffer solution (pH 7.4) and kept at 4°C. Tris-HCL buffer was widely used as the solvent of the protein, which could prevent the dramatic fluctuation of PH, thus preventing the denaturation of the protein which could create an approximate physiological condition. The concentration was measured by Uv-visible absorption spectrum, to which the extinction coefficient at 280 nm of 36,600 L.mol^−1^.cm^−1^ was applied. AgInZnS nanoparticles were synthesized base on previous research. The oily AIZS QDs were transferred by graphene oxide into red-emission water soluble quantum dots. All the experiments used ultrapure water.

### Synthesis of AIZS QDs

The synthesis of high quality water-soluble AIZS QDs is an important factor in the research, which enabled it to be further applied in biological detection. The main synthesis process was as follows. First, 0.1 mM indium acetate, 0.1 mM silver nitrate, 0.1 mM oleic acid, 2 mM double chlorobenzene trichloroethane, 40 ml trioctylphosphine, and 4 mM octadecene, respectively, were added to three neck flasks heated to 85°C for 30 min. Next, 0.1 mM sulfur was added into the ODE and oleic acid, after which they were gradually added into a three-neck flask. After 2 min, 0.1 mM zinc was dissolved in the ODE and oleic acid and gradually added into the reaction solution. At the same time, the solution was quickly heated to 130°C, and 10 drops of zinc stearate was then added, and the ODE mixture was annealed for 10 min. Lastly, the mixture left to cool to room temperature. Oil-soluble AIZS QDs are obtained by centrifugation after dropping ethanol. Based on the synthesis of oil-soluble AIZS QDs, the AIZS-GO QDs composite nanomaterials can be further synthesized by micro-emulsification. First, GO is modified with oleylamine as the emulsifier, and then GO-OAM is precipitated after cleaning. Finally, GO-OAM and oil-soluble AIZS QDs are mixed in chloroform, then the pure AIZS-GO QDs are obtained through a series of processes.

### Fluorescence Spectroscopy

All fluorescence spectra were obtained by Cary Eclipse spectrofluorometer (Varian, USA), which was equipped with a 150 W xenon lamp and a thermostatic water bath. Quartz cells (1 cm path-length) were applied to all measurements. At various temperatures (298 K, 303 K, 308 K), the fluorescence emission spectra were obtained with a wavelength range of 300–480 nm and excitation at 285 nm as titration of QDs was used in HSA. Excitation and emission slit widths were then set to 5.0 nm and 10.0 nm. The averages of the three scans were reflected in the spectra. To begin with, 2.8 ml of 1 × 10^−6^ mol/L HSA solution was added to a 10 mm quartz cell and 5 ul AIZS-GO QDs of different concentrations was subsequently added to the HSA each time, which meant that a series of HSA concentrations were obtained to determine the quenching capacity.

### Uv-Visible Absorption Spectroscopy

Uv-visible absorption spectrum was recorded by Cary 60 Uv-visible spectrophotometer (Varian, USA), which was equipped with 10 mm quartz cells. The recorded wavelength ranged from 220 to 440 nm at 298 K. The dissimilitude between AIZS-GO QDs-HSA and AIZS-GO QDs was acquired by the Uv-visible absorption spectrum of AIZS-GO QDs-HSA, which put AIZS-GO QDs as the reference.

### CD Spectroscopy

CD spectrum was recorded by MOS-450 CD spectropolarimeter (Bio-Logic, France), which was equipped with 10 mm quartz cells. The CD spectrum ranged from 190 to 250 nm and the acquisition duration was set at 2 s. Each spectrum was corrected by 0.05 M Tris-HCl buffer solution. The results were the average of the three scans. As reported in the literature, the tris buffer system was widely used as the solvent of protein, which could prevent the dramatic fluctuation of PH, thus preventing the denaturation of protein, which could create an approximate physiological condition (Chuang and Otagiri, 2002; Hu et al., 2005; Zhang et al., 2008; Feroz et al., 2012; Lin et al., 2017, 2019b). So, we used the tris buffer system as the environment for the protein binding assay *in vitro*.

### FT-IR Spectroscopy

Fourier transform infrared (FT-IR) spectrum was measured by Nicolet iS5 spectrometer (Thermo, USA). All FT-IR spectra were obtained in the range of 1,300–1,800 cm^−1^ with the resolution ratio of 4 cm^−1^. Tris-HCl solution as the reference solution was used to ensure the spectra of HSA and AIZS-GO QDs-has were not influenced.

## Results and Discussion

### Characterization of AgInZnS-GO QDs

**Figure 1:**
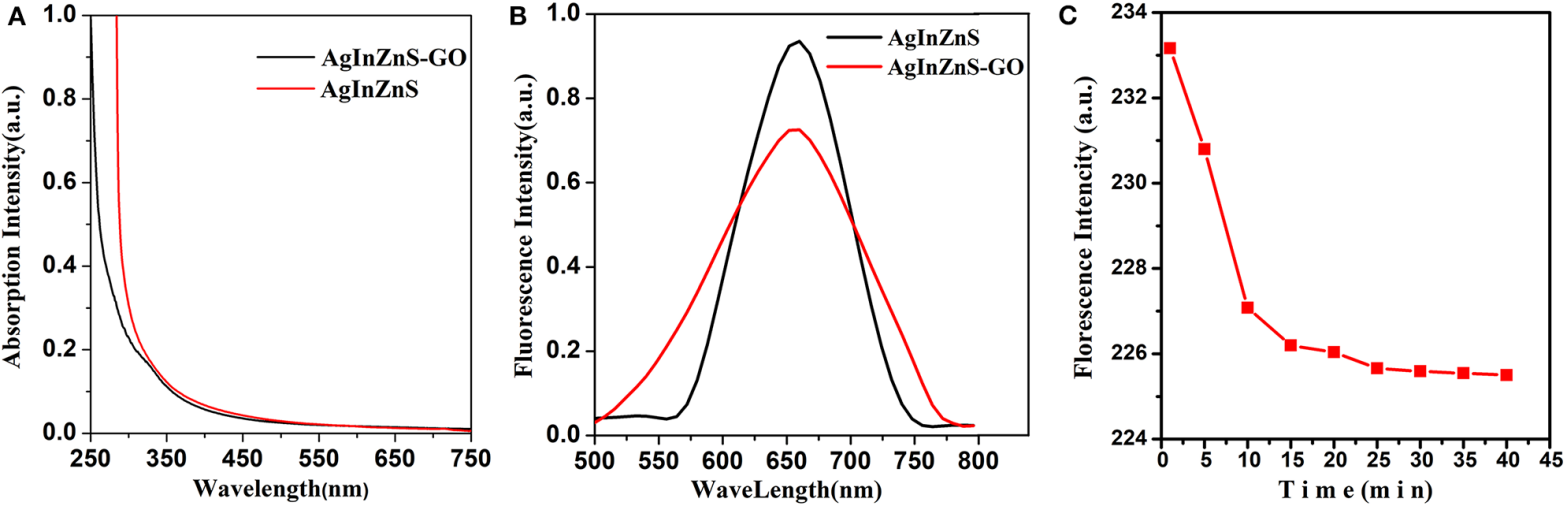
**(A)** Uv-visible absorption of AIZS QDs and AIZS-GO QDs; **(B)** the fluorescence spectra of AgInZnS QDs and AgInZnS-GO QDs; **(C)** the influence of reaction time on the interaction between AIZS-GO QDs and HSA.

The typical Uv-visible absorption of AIZS QDs and AIZS-GO QDs can be seen in Figure 1A. Both had strong absorptivity but had no obvious absorption peak. AIZS-GO QDs had high water solubility while AIZS QDs had high oil solubility. From Figure 1B, we can see the fluorescence spectra of AgInZnS QDs and AgInZnS-GO QDs, which demonstrates that AIZS QDs and AIZS-GO QDs exhibit obvious and almost symmetrical emission spectrums with excitation wavelengths of 370 nm. The emission wavelength of AIZS QDs was about 570–750 nm, while the emission wavelength of AIZS-GO QDs was about 500–780 nm. The spectrums were symmetric, indicating that they were homogenous.

### Effects of Reaction Time

By observing the reaction time influence on the florescence intensity of the AIZS-GO QDs and HSA system, the extent of interaction in the system could be researched better. As shown in Figure 1C, in the first 20 min during the reaction process, the fluorescence intensity of the system reduced along with the time. That was because AIZS-GO QDs had an obvious fluorescence quenching effect on HSA and it was a static quenching. In the early stage of the static quenching, AIZS-GO QDs could combine with the fluorescent molecules which were in the ground state, so as to generate the new compound which had no fluorescence characteristics. That is to say, the photons were not emitted in the new compound, which resulted in the dramatic decline of the fluorescence intensity in the first 20 min. After 25 min, the number of new compounds formed by the combination between AIZS-GO QDs and HSA was stable, and the fluorescence intensity of the system was approximately unchanged. All experiment data should therefore be tested after 25 min, when the AIZS-GO solution titrated.

### Fluorescence Quenching of HSA by QDs

**Figure 2:**
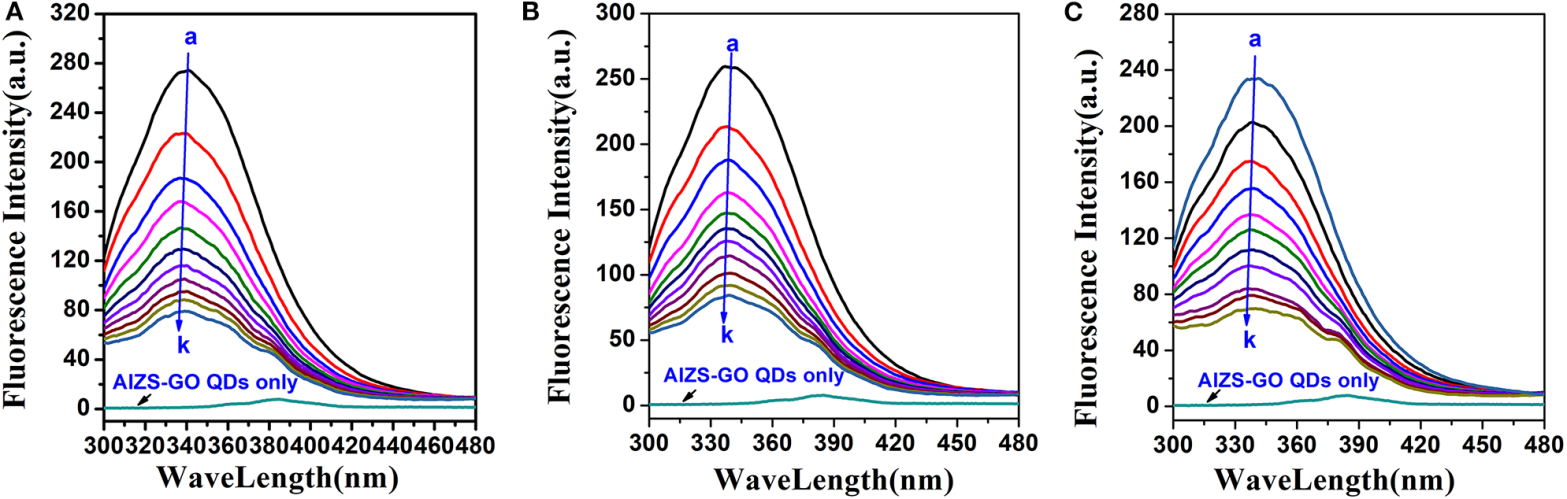
Influence of AIZS-GO QDs on HAS fluorescence intensity at diverse temperatures. **(A)** 298 K, **(B)** 303 K, **(C)** 308 K. c(HSA) = 1.0 × 10^−6^*molL*^−1^, c(AIZS-GO QDs): (a-k): 0, 1, 2, 3, 4, 5, 6, 7, 8, 9, 10 × 10^−6^*molL*^−1^ pH = 7.4, λex = 285 nm.

AIZS-GO QDs are able to effectively quench the fluorescence intensity of HSA. Figure 2 shows the influence of AIZS-GO QDs on HSA fluorescence intensity. It obviously illustrated that the fluorescence intensity decreased bit by bit when the concentration of AIZS-GO QDs increased at diverse temperatures (298 K, 303 K, 308 K). At the same time, Figure 2 shows that AIZS-GO QDs had a weak fluorescence peak when the excitation wavelength was 285nm, but the fluorescence peak did not appear at 340 nm where the fluorescence peak of AIZS-GO QDs and the HSA system appeared. Furthermore, the fluorescence peak value of AIZS-GO QDs was very low. Therefore, the fluorescence of AIZS-GO QDs will not affect the fluorescence spectrum of HSA. We did not therefore correct the fluorescence data for inner filter effect.

The fluorescence quenching mechanism could be distributed into dynamic quenching and static quenching. Quencher and fluorescence materials generated no fluorescing complex in static quenching, which could result in the decrease of the fluorescence intensity. Dynamic quenching was caused by quencher and fluoresce material molecules colliding at the excited state. Quenching mechanisms could be distinguished by quenching constants at various temperatures (Ghali, 2010; Lacerda et al., 2010). Higher temperatures lead to larger diffusion coefficients and larger masses of collisional quenching. As a result, higher temperatures could also increase the dynamic quenching constants. On the contrary, higher temperatures could not only bring about stability of decreased complexes, but also decreased the static quenching constants (Brown and Royer, 1997; Dzagli et al., 2010). The fluorescence quenching is able to be ordinarily determined by Stern-Volmer equation.

(1) $$\begin{array}{l} \frac{F_{0}}{F}=1+K_{q}\tau_{0}\left[Q\right]=1+K_{SV}\left[Q\right] \end{array}$$

where *F*_0_ and *F* are steady-state of fluorescence intensities before and after adding quencher (AIZS-GO QDs solution), respectively. *K*_*q*_ is the quenching rate constant of bimolecular, *K*_*SV*_ is the Stern-Volmer quenching constant, [*Q*] is the concentration of quenching agent.

**Figure 3:**
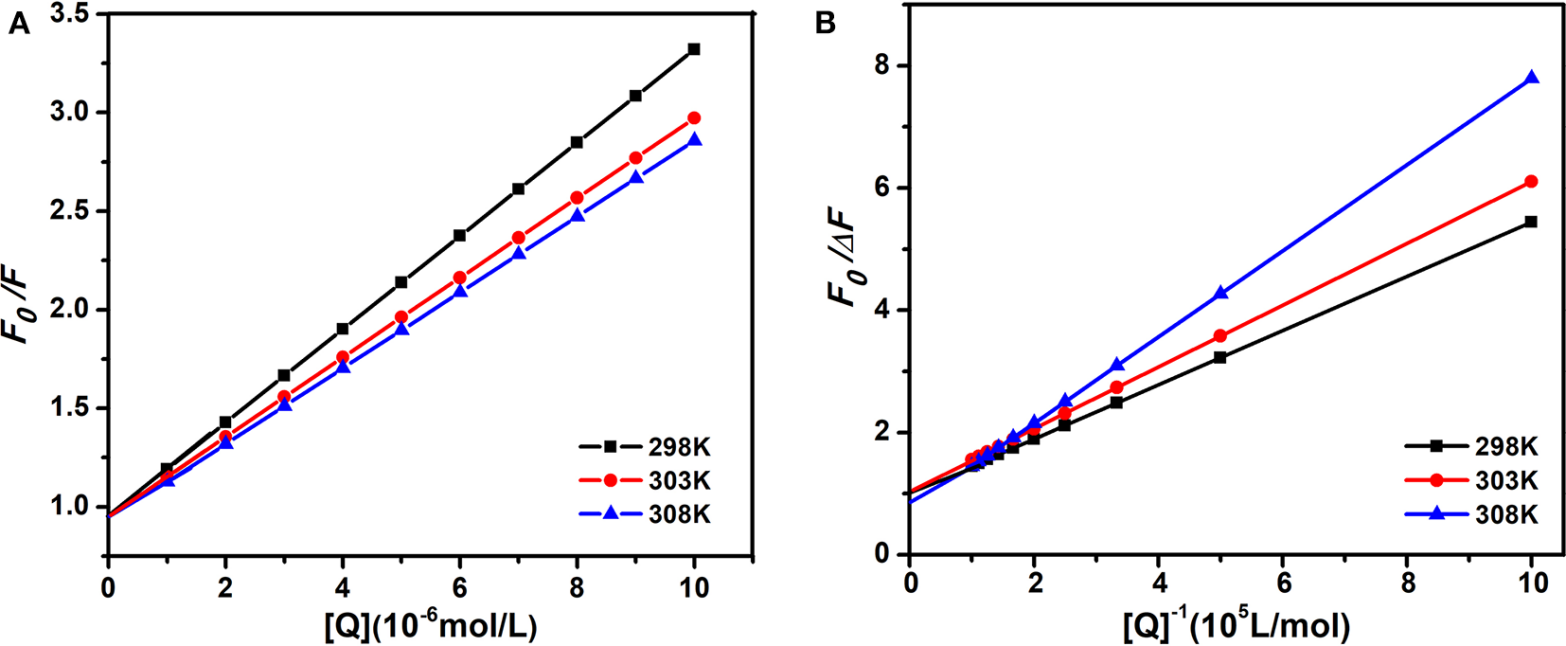
The Stern-Volmer and amendatory Stern-Volmer curves of AIZS-GO QDs with HSA at diverse temperatures. **(A)** Stern-Volmer, **(B)** the amendatory Stern-Volmer.

Therefore, the above equation could determine *K*_*SV*_ by linear regression of a plot of *F*_0_/*F* against [Q]. Figure 3A shows the graphs of Stern-Volmer equation at diverse temperatures. And the Stern-Volmer quenching constant *K*_*SV*_ values are listed in Table 1.

**Table 1:** Stern-Volmer quenching constant *K*_*SV*_, amendatory Stern-Volmer effective quenching constant *K*_*a*_ and the correlation coefficient R for the interaction between AIZS-GO QDs and HSA at diverse temperatures.

**T(K)**	***K*_sv_(10^5^*Lmol*^−1^)**	**R**	***K*_s_(10^5^*Lmol*^−1^)**	**R**
298	2.230	0.9999	2.147	0.9993
303	1.922	0.9996	1.970	0.9995
308	1.829	0.9994	0.999	0.9998

In order to determine whether it was dynamic quenching or static quenching, we explored the quenching mechanism of AIZS-GO QDs and HSA systems by studying fluorescence quenching under different temperatures. It was obvious that the Stern-Volmer quenching constant *K*_*SV*_ reduced when the temperature increased, which implied that the quenching mechanism was static quenching.

Moreover, the quenching information was further verified by the amendatory Stern-Volmer equation (Liang et al., 2008; Asha Jhonsi et al., 2009; Sun et al., 2015).

(2) $$\begin{array}{l} \frac{F_{0}}{\Delta F}=\frac{F_{0}}{F_{0}-F}=\frac{1}{f_{a}K_{a}}\frac{1}{\left[Q\right]}+\frac{1}{f_{a}} \end{array}$$

where Δ*F* is a variation of fluorescence intensity, *f*_*a*_ is a proportion of accessible fluorescence. *K*_*a*_ is an effective quenching constant. The dependence of *F*_0_/Δ*F* on the reciprocal value of the quenching agent concentration [Q]^−1^ was linear with the slope, equaling the value of ${\left(f_{a}K_{a}\right)}^{-1}$ and the ordinate equaling the value of ${f_{a}}^{-1}$. Figure 3B shows the graphs of the amendatory Stern-Volmer equation at diverse temperatures.

We found that the effective quenching constant *K*_*a*_ reduced when temperature increased in Table 1. And it was same as the change of *K*_*SV*_, which further implied the static quenching mechanism of AIZS-GO QDs and the HSA system.

Moreover, the *K*_*a*_ values varied slightly, indicating that the combination between AIZS-GO QDs and HSA is stronger, which is a result of the synergistic forces among ions. Meanwhile, these demonstrate that QDs will stay in the blood plasma longer and will be more difficult clear away.

**Figure 4:**
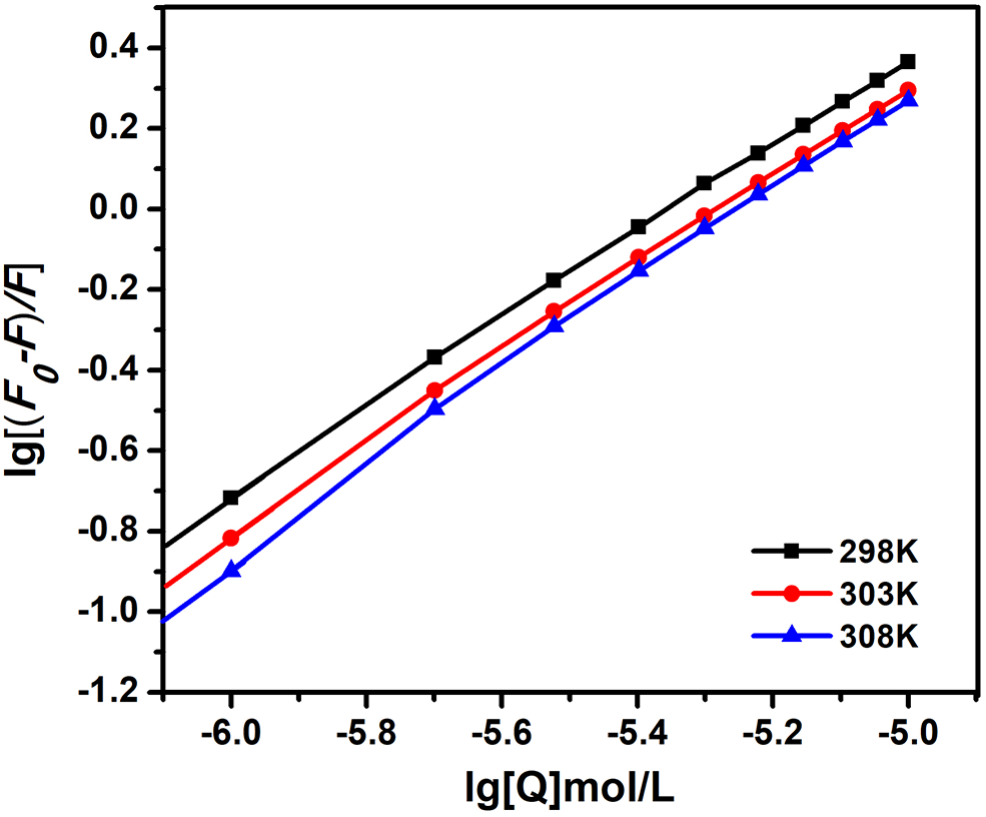
The double log plot of AIZS-GO QDs with HSA at diverse temperatures.

When quantum dots bind with HSA, molecules were in a state of partial equilibrium. This relationship can be calculated using the following equation (Huang et al., 2012; Tabassum et al., 2012).

(3) $$\begin{array}{l} \mathrm{lg}\frac{F_{0}-F}{F}=lgK_{A}\;+\;nlg\left[Q\right] \end{array}$$

where *K*_*A*_ is the binding constant and *n* is the number of binding points in the interaction between AIZS-GO QDs and HSA at diverse temperatures. Figure 4 shows graphs of the double log equation at diverse temperatures. The binding constant *K*_*A*_ values and the number of binding points *n* are listed in Table 2. From the results, *n* was close to 1, which indicates that there was at least one binding point in the interaction between AIZS-GO QDs and HSA. The binding constant is importance to understand the distribution of AIZS-GO solution. The binding constant increased with rising temperatures, meaning that the complex produced in the reaction became more stable. From the above analysis, we could form a site binding model to judge the reaction degree of protein and quenching agent. That was why we studied the other temperatures condition in this study.

**Table 2:** The binding constant *K*_*A*_, numbers of binding site n and the correlation coefficient R for the interaction between AIZS-GO QDs and HSA at diverse temperatures.

**T(K)**	***K*_sv_(10^5^*Lmol*^−1^)**	***n***	**R**
298	5.695	1.076	0.9992
303	6.490	1.101	0.9988
308	10.942	1.150	0.9973

### Combination Between QDs and HSA

The combination power between extraneous parts and HSA mainly involve hydrophobic, van der Waals, and hydrogen bonds (Sudlow et al., 1976). Based on plenty of experimental data and by analyzing results, Ross and Surbamanian concluded that the interaction forces were associated with a magnitude of thermodynamic parameters, containing enthalpy (ΔH) and entropy (ΔS). The thermodynamic parameters can be described by van't Hoff's equation (Koegler et al., 2012).

(4) $$\begin{array}{l} \ln K_{A}=-\frac{H}{RT}+\frac{S}{R}\; \end{array}$$

(5) $$\begin{array}{l} G=-RTlnK_{A}=H-TS \end{array}$$

where *K*_*A*_ is the binding constant at settled temperature T, R is the gas constant, and Δ*G* is free energy. We could draw the linear fitting plot of ln *K*_*A*_ against $\frac{1}{RT}$. The values of Δ*H* and Δ*S* were acquired from the slope and intercept of the plot. The values are shown in Table 3. The values of Δ*H* and Δ*S* were both less than zero demonstrating that hydrogen bonds and van der Waals' forces made great contributions to the interaction of AIZS-GO QDs and HSA. The values of Δ*G* were negative demonstrating that the interaction is a spontaneous process.

**Table 3:** The thermodynamic parameters of AIZS-GO QDs and HSA system at diverse temperatures.

**T(K)**	**ΔH(KJmol^**−1**^)**	**ΔG (KJmol^**−1**^)**	**ΔS (Jmol^**−1**^K^**−1**^)**
298		−30.723	
303	−66.417	−30.124	−119.78
308		−29.525	

### UV-Vis Absorption Spectra

Furthermore, the distinction of Uv-visible absorption spectrum could identify the fluorescence quenching mechanism. Dynamic quenching only affects exited states of molecules, and there was no distinction in the absorption spectrum. But the complex generation in the process of static quenching disturbed the absorption spectrum (Gelamo et al., 2002; Hu et al., 2010).

**Figure 5:**
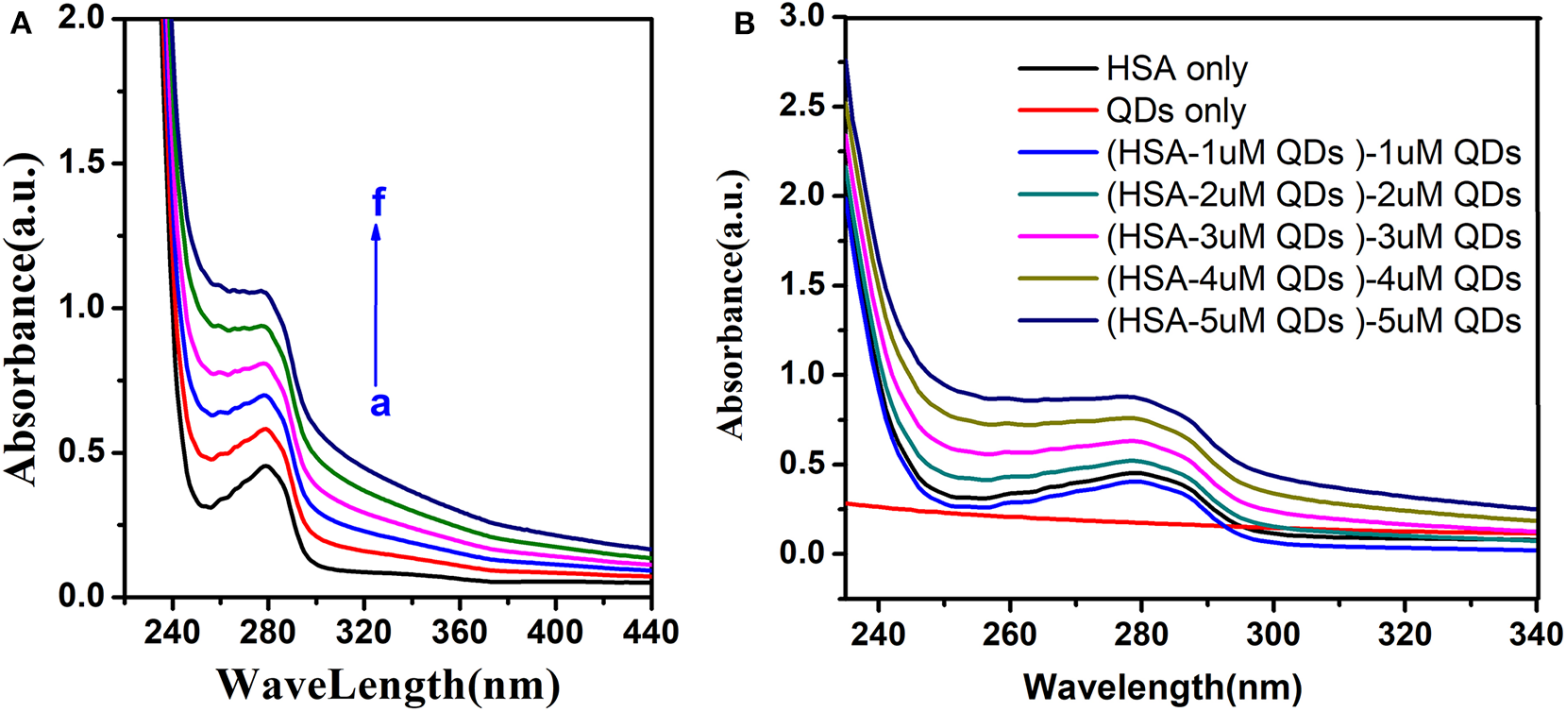
**(A)** Uv-visible absorption spectra of HSA (T = 298 K) in different concentration of AIZS-GO QDs. c(HSA) = 1.0 × 10^−6^*molL*^−1^, c(AIZS-GO QDs): (a-f): 0, 1, 2, 3, 4, 5 × 10^−6^*molL*^−1^, pH = 7.4. **(B)** is the Uv-visible absorption spectra of QDs, HSA and the distinction of HSA-QD and QDs.

We could see that the Uv-visible absorption spectrum of HSA was significantly altered by adding different concentrations of AIZS-QDs in Figure 5A. Figure 5B shows that AIZS-GO QDs have strong absorptivity, and the absorption spectrum between the AIZS-GO QDs-HSA system and AIZS-GO illustrated that changes in the absorption spectrum of has, after instilling AIZS-GO, was not influenced by the absorbency of quantum dots. This further confirmed the static quenching mechanism.

### Conformation Alteration of HSA by QDs

**Figure 6:**
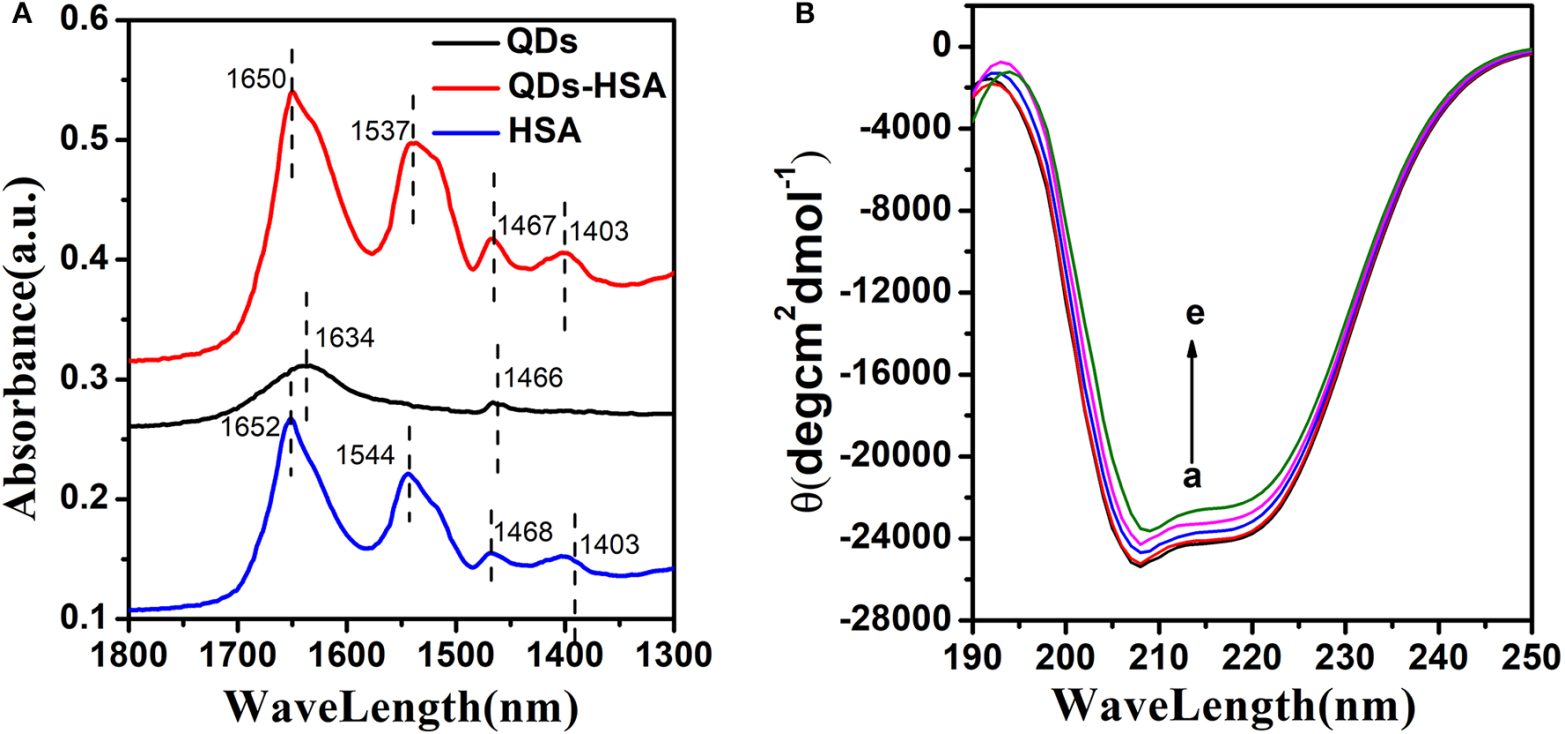
**(A)** FT-IR spectra of HSA, AIZS-GO QDs, and AIZS-GO QDs-HSA system. **(B)** CD spectra of HSA with different concentration AIZS-GO QDs. c(HSA) = 1.0 × 10^−6^*molL*^−1^, c(AIZS-GO QDs): (a-e): 0, 2, 4, 6, 8 × 10^−6^*molL*^−1^. pH = 7.4.

FT-IR spectroscopy can effectively analyze the alteration of the structure of HSA (Ding et al., 2011; Hemmateenejad et al., 2015). Figure 6A shows that HSA had two primary amide bands of about 1652 cm^−1^ (C=O stretch) and 1,544 cm^−1^(C-N stretch, N-H bending mode). QDs had two primary absorption bands of about 1,634 cm^−1^ (C=O stretch) and 1,466 cm^−1^ (C-H stretch), too. Furthermore, the absorption spectrum of AIZS-GO QDs-HSA had a distinct wavelength movement (from 1,652–1,650 to 1,544–1,537 cm^−1^) when it was compared with HSA′s. These results demonstrate that QDs interact with groups on the surface of HSA that alter HSA's structure.

CD spectroscopy can research the conformation of HSA quantitatively (Nordén and Tjerneld, 1982; Venyaminov and Woody, 1999). Figure 6B shows that HSA had two negative absorption points of about 208, 220 nm. It implies that the α-helical structure existed in HSA. This phenomenon was owed to the n → π^*^ and π → π^*^ electron transfer to the peptide's bonds of the α-helica. Meanwhile, the diversity between the HSA and AIZS-GO QDs-HSA system demonstrates the cause of the conformational alteration of HSA. CD spectra can be described using following equation.

(6) $$\begin{array}{l} {MRE}_{208nm}=\frac{ObservedCD\left(mdeg\right)}{cnl\times 10} \end{array}$$

(7) $$\begin{array}{l} \alpha -helix\left(\%\right)=\frac{-{MRE}_{208nm}-4000}{33000-4000}\times 100\; \end{array}$$

Where MRE_208nm_ is the mean residue ellipticity at 208 nm, c is the concentration of HSA, n is the number of amino acid residues in HSA (*n* = 585) and l is the path length. It was calculated that approximately 54.52% of the α-helix existed in HSA, which implied that HSA remained α-helix structure. In addition, when AIZS-GO QDs exist, the α-helix would decrease to 50.35%, which demonstrates more alterations in the structure and that surface coverage of HSA is reduced after interacting with AIZS-GO QDs. The α-helix content of HSA has an effect on the bioactivity of HSA.

All FT-IR spectra and CD spectra results demonstrate that AIZS-GO QDs bind stronger with HSA, which leads to major conformation alterations of HSA.

### Cytotoxicity of QDs

**Figure 7:**
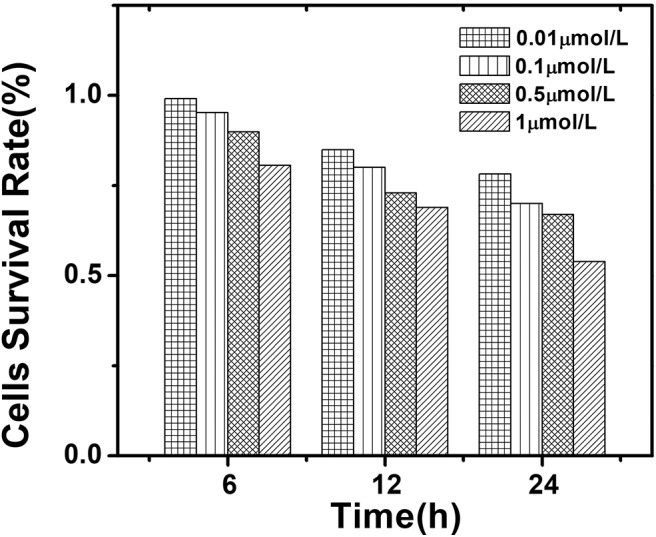
The influence of different concentrations of AIZS-GO QDs and different incubation time on the survival rate of hepatocellular carcinoma Hep-G2 cells.

The quantum dots in this work were nearly non-toxic. When AIZS-GO QDs are gradually applied to live experiments, their toxicity always needs to be further researched systematically. In order to use AIZS-GO QDs to label liver cancer cells, the cck-8(Cell Counting Kit-8) analytical method was used to test the survival rate of hepatocellular carcinoma Hep-G2 cells and to determine the cytotoxicity of AIZS-GO QDs in the experiment. The results were the average of five tests. The cytotoxicity of AIZS-GO QDs was analyzed by using quantum dots with different concentrations (1,10,50,100 μ*mol*/*L*) and different action times (6, 12, 24 h). The cell survival rate is shown in Figure 7.

Figure 7 shows that the damage to cells was the greatest when the concentration of AIZS-GO QDs was 10^−6^ *mol*/*L*. After 24 h of culture, cell survival rate was only 53.87%. With the increased action time, the cell survival rate gradually decreased at different concentrations. When the concentration of AIZS-GO QDs was 10^−8^*mol*/*L* and the action time was 24 h, the cell survival rate was still 78.15%, indicating that the effect of AIZS-GO QDs on cell activity was not significant at the appropriate concentration. The results also implied that AIZS-GO QDs could affect the cells' survival rate by cultivating with them for too long. Moreover, when the concentration of AIZS-GO QDs exceeded a certain range, the apoptosis rate of cells could increase.

Table 4 shows the comparison in cytotoxicity of different quantum dots of the other reported work. It was shown that the synthesized AIZS-GO QDs had greatly lowered cytotoxicity than cadmium quantum dots which contained heavy metal ions. It is beneficial for us to study the cytotoxicity of AIZS-GO QDs in normal cells in the future.

**Table 4:** Cytotoxicity comparison of different quantum dots after 6 h.

**Quantum dots**	**Concentration of QDs**	**Cells**	**Cells survival rate**	**Reference**
NAC-CdTe QDs	20 nM	Hepatocytes	75.38%	Wang et al., 2019
CdSe QD-polymerNPs	10 nM	HaCaT cells	71.84%	Ando et al., 2016
AIZS-GO QDs	10 nM	Hep-G2 cells	99.15%	This work
AIZS-GO QDs	100 nM	Hep-G2 cells	95.23%	This work

## Conclusions

In summary, the interaction between AIZS-GO QDs and HSA was researched by fluorescence spectra, Uv-visible absorption spectra, FT-IR spectra, and CD spectra. AIZS-GO QDs then closely bind to HSA and form a complex, and hydrogen bonds and van der Waals' forces have made major contributions to this binding process. The Stern-Volmer quenching constants and thermodynamic parameters were determined by fluorescence information. The results demonstrate that the fluorescence intensity of HSA can be quenched by AIZS-GO QDs. The FT-IR spectra and CD spectra further accounted for the structure alterations of HSA when AIZS-GO QDs existed. It means that AIZS-GO QDs can influence the bioactivity of HSA. All the results are conducive to understanding the interaction between AIZS-GO QDs and HSA. Besides HSA, we also found that AIZS - GO QDs could combine with the lysozyme which is a kind of protein in the blood. After AIZS - GO QDs combined with the lysozyme, however, the fluorescence characteristic, Uv-visible absorption spectroscopy, and the conformation of the lysozyme were obviously distinct from the corresponding performance of HSA. It also implied that after AIZS - GO QDs combined with HSA, the performance of HSA had specificity. In the future, specific antibodies and antigens can be connected on the surface of quantum dots through this binding mechanism to make bioluminescence probes to detect and label corresponding proteins or tumor cells. It can also further promote the development of medical diagnostics and medical research. Compared with the other substitutes, AIZS-GO QDs have high luminescence efficiency, low preparation costs, and strong fluorescence performance. Most importantly, the advantage is that AIZS-GO QDs are non-toxic because they do not contain heavy metal ions which are toxic. In other words, AIZS-GO QDs have more potential, than other substitutes, to be applied in biological fields for medical diagnosis and research. The experimental results show that AgInZn-GO water soluble quantum dots have good biocompatibility, which can be combined with proteins to form new compounds and have no cytotoxicity and biological practicability. It provides an important basis for the combination of quantum dots and specific proteins and fluorescent labeling (Zhou et al., 2019).

## Data Availability Statement

All datasets generated for this study are included in the article/supplementary material.

## Author Contributions

CS, HL, LW, and ZP: methodology, formal analysis, investigation, data curation, and writing—original draft. XL and XZ: conceptualization, validation, writing—review and editing, supervision, project administration, and funding acquisition. XT, SX, MS, and LJ resources and writing—original draft.

## Conflict of Interest

The authors declare that the research was conducted in the absence of any commercial or financial relationships that could be construed as a potential conflict of interest.

## References

[B1] Alivisatos A. P. (1996). Semiconductor clusters, nanocrystals, and quantum dots. Science 271, 933–937. 10.1126/science.271.5251.933

[B2] Allen P. M. Bawendi M. G. (2008). Ternary I-III-VI quantum dots luminescent in the red to near-infrared. J. Am. Chem. Soc. 130, 9240–9241. 10.1021/ja8036349 18582061 PMC2877636

[B3] Ando M. Horie M. Akazawa-Ogawa Y. Hagihara Y. Murase N. Shigeri Y. (2016). Cytotoxicity of CdSe-based quantum dots incorporated in glass nanoparticles evaluated using human keratinocyte HaCaT cells. Biosci. Biotechnol. Biochem. 80, 210–213. 10.1080/09168451.2015.1069702 26214259

[B4] Asha Jhonsi M. Kathiravan A. Renganathan R. (2009). Spectroscopic studies on the interaction of colloidal capped CdS nanoparticles with bovine serum albumin. Colloids Surfaces B Biointerfaces 72:167. 10.1016/j.colsurfb.2009.03.030 19410435

[B5] Brown M. P. Royer C. (1997). Fluorescence spectroscopy as a tool to investigate protein interactions. Curr. Opin. Biotechnol. 8, 45–49. 10.1016/S0958-1669(97)80156-5 9013650

[B6] Bruchez M. Moronne M. Gin P. Weiss S. Alivisatos A. P. (1998). Semiconductor nanocrystals as fluorescent biological labels. Science 281:2013. 10.1126/science.281.5385.2013 9748157

[B7] Carter D. C. He X. M. Munson S. H. Twigg P. D. Gernert K. M. Broom M. B.. (1989). Three-dimensional structure of human serum albumin. Science 244, 1195–1198. 10.1126/science.2727704 2727704

[B8] Cassette E. Pons T. Bouet C. (2010). Synthesis and characterization of near-infrared CuInSe/ZnS Core/Shell quantum dots for *in vivo* imaging. Chem. Mater. 22, 6117–6124. 10.1021/cm101881b

[B9] Chan W. C. Nie S. (1998). Quantum dot bioconjugates for ultrasensitive nonisotopic detection. Science 281, 2016–2018. 10.1126/science.281.5385.2016 9748158

[B10] Chuang V. T. Otagiri M. (2002). How do fatty acids cause allosteric binding of drugs to human serum albumin? Pharm. Res. 19, 1458–1464. 10.1023/A:1020496314081 12425462

[B11] Dabbousi B. O. Rodriguez-Viejo J. Mikulec F. V. Mattoussi H. Ober R. Jensen K. F. (1997). ChemInform abstract: (CdSe) ZnS core—shell quantum dots: synthesis and characterization of a size series of highly luminescent nanocrystallites. Cheminform 101, 9463–9475. 10.1021/jp971091y

[B12] Ding L. Zhou P. J. Li S. Q. Shi G. Y. Zhong T. Wu M. (2011). Spectroscopic studies on the thermodynamics of L-cysteine capped CdSe/CdS quantum dots–BSA interactions. J. Fluoresc. 21, 17–24. 10.1007/s10895-010-0685-2 20593228

[B13] Dong A. Huang P. Caughey W. S. (1990). Protein secondary structures in water from second-derivative amide I infrared spectra. Biochemistry 29, 3303–3308. 10.1021/bi00465a022 2159334

[B14] Dzagli M. Canpean V. Iosin M. Mohou M. A. Astilean S. (2010). Study of the interaction between CdSe/ZnS core-shell quantum dots and bovine serum albumin by spectroscopic techniques. J. Photochem. Photobiol. A Chem. 215, 118–122. 10.1016/j.jphotochem.2010.08.008

[B15] Farid M. M. Goudini L. Piri F. Zamani A. Saadati F. (2016). Molecular imprinting method for fabricating novel glucose sensor: Polyvinyl acetate electrode reinforced by MnO2/CuO loaded on graphene oxide nanoparticles. Food Chem. 194:61. 10.1016/j.foodchem.2015.07.128 26471527

[B16] Feroz S. R. Mohamad S. B. Bujang N. Malek S. N. Tayyab S. (2012). Multispectroscopic and molecular modeling approach to investigate the interaction of flavokawain B with human serum albumin. J. Agri. Food Chem. 60, 5899–5908. 10.1021/jf301139h 22624666

[B17] Figge J. Rossing T. H. Fencl V. (1991). The role of serum proteins in acid-base equilibria. J. Lab. Clin. Med. 117, 453–467. 2045713

[B18] Fu Y. Lakowicz J. R. (2006). Enhanced fluorescence of Cy5-Labeled DNA tethered to silver island films: fluorescence images and time-resolved studies using single-molecule spectroscopy. Analyt. Chem. 78, 6238–6245. 10.1021/ac060586t 16944907 PMC6830066

[B19] Gao Y. Zou X. Zhao J. X. Li Y. Su X. (2013). Graphene oxide-based magnetic fluorescent hybrids for drug delivery and cellular imaging. Colloids Surfaces B Biointerfaces. 112, 128–133. 10.1016/j.colsurfb.2013.07.020 23973670

[B20] Gelamo E. L. Silva C. H. Imasato H. Tabak M. (2002). Interaction of bovine (BSA) and human (HSA) serum albumins with ionic surfactants: spectroscopy and modelling. Biochim. Biophys. Acta 1594, 84–99. 10.1016/S0167-4838(01)00287-4 11825611

[B21] Ghali M. (2010). Static quenching of bovine serum albumin conjugated with small size CdS nanocrystalline quantum dots. J. Luminescence 130, 1254–1257. 10.1016/j.jlumin.2010.02.034

[B22] Hamanaka Y. Ogawa T. Tsuzuki M. (2011). Photoluminescence properties and its origin of AgInS2 quantum dots with chalcopyrite structure. J Phys Chem. C 115, 1786–1792. 10.1021/jp110409q

[B23] Hemmateenejad B. Shamsipur M. Samari F. Rajab H. R. (2015). Study of the interaction between human serum albumin and Mn-doped ZnS quantum dots. J. Iran. Chem. Soc. 12, 1–10. 10.1007/s13738-015-0647-3

[B24] Hu Y. J. Liu Y. Pi Z. B. Qu S. S. (2005). Interaction of cromolyn sodium with human serum albumin: a fluorescence quenching study. Bioorg. Med. Chem. 13, 6609–6614. 10.1016/j.bmc.2005.07.039 16126393

[B25] Hu Y. J. Yu O. Y. Bai A. M. Li W. Liu Y. (2010). Investigation of the interaction between ofloxacin and bovine serum albumin: spectroscopic approach. J. Solut. Chem. 39, 709–717. 10.1007/s10953-010-9527-8

[B26] Huang S. Qiu H. Lu S. Zhu F. Xiao Q. (2012). Study on the molecular interaction of graphene quantum dots with human serum albumin: combined spectroscopic and electrochemical approaches. J. Hazard Mater. 285, 18–26. 10.1016/j.jhazmat.2014.11.019 25462867

[B27] Jiang Y. Tang N. Zhou C. Han Z. Qu H. Duan X. (2018). A chemiresistive sensor array from conductive polymer nanowires fabricated by nanoscale soft lithography. Nanoscale 10, 20578–20586. 10.1039/C8NR04198A 30226241

[B28] Koegler P. Clayton A. Thissen H. Santos G. N. C. Kingshott P. (2012). The influence of nanostructured materials on biointerfacial interactions. Adv. Drug Deliv. Rev. 64, 1820–1839. 10.1016/j.addr.2012.06.001 22705547

[B29] Lacerda S. H. Park J. J. Meuse C. Pristinski D. Becker M. L. Karim A.. (2010). Interaction of gold nanoparticles with common human blood proteins. ACS Nano 4, 365–379. 10.1021/nn9011187 20020753

[B30] Leckband D. (2000). Measuring the forces that control protein interactions. Ann. Rev. Biophys. Biomol. Struct. 29, 1–26. 10.1146/annurev.biophys.29.1.1 10940241

[B31] Li J. Parisi J. Kolny-Olesiak J. (2015). Synthesis of CuInS2-ZnS alloyed nanorods and hybrid nanostructures. MRS Proc. 1780:mrss15-2136281. 10.1557/opl.2015.771

[B32] Liang J. Cheng Y. Han H. (2008). Study on the interaction between bovine serum albumin and CdTe quantum dots with spectroscopic techniques. J. Mol. Struct. 892, 116–120. 10.1016/j.molstruc.2008.05.005

[B33] Lin S. Bai X. Wang H. Wang H. Song J. Huang K.. (2017). Roll-to-roll production of transparent silver-nanofiber-network electrodes for flexible electrochromic smart windows. Adv. Mater. 29:1703238. 10.1002/adma.201703238 28892194

[B34] Lin S. Liu J. Li W. Wang D. Huang Y. Jia C.. (2019a). A flexible, robust, and gel-free electroencephalogram electrode for noninvasive brain-computer interfaces. Nano Lett. 19, 6853–6861. 10.1021/acs.nanolett.9b02019 31454250

[B35] Lin S. Wang H. Zhang X. Wang D. Zu D. Song J. (2019b). Direct spray-coating of highly robust and transparent Ag nanowires for energy saving windows. Nano Energy 62, 111–116. 10.1016/j.nanoen.2019.04.071

[B36] Niemeyer C. M. (2001). Nanoparticles, proteins, and nucleic acids: biotechnology meets materials science. Angew. Chem. Int. Ed. 40, 4128–4158. 10.1002/1521-3773(20011119)40:22<4128::AID-ANIE4128>3.0.CO;2-S 29712109

[B37] Nirmal M. Murray C. B. Bawendi M. G. (1994). Fluorescence-line narrowing in CdSe quantum dots: Surface localization of the photogenerated exciton. Phys. Rev. B Condensed Matter. 50:2293. 10.1103/PhysRevB.50.2293 9976446

[B38] Nordén B. Tjerneld F. (1982). Structure of methylene blue-DNA complexes studied by linear and circular dichroism spectroscopy. Biopolymers 21, 1713–1734. 10.1002/bip.360210904 7126754

[B39] Ragab A. E. Gadallah A. S. Mohamed M. B. Azzouz I. M. (2014). Photoluminescence and upconversion on Ag/CdTe quantum dots. Optics Laser Technol. 63, 8–12. 10.1016/j.optlastec.2014.03.006

[B40] Sheng Y. Tang X. Peng E. Xue J. (2012). Graphene oxide based fluorescent nanocomposites for cellular imaging. J. Mater. Chem. B 1, 512–521. 10.1039/C2TB00123C 32260822

[B41] Shiang J. J. Kadavanich A. V. Grubbs R. K. Alivisatos A. P. (1995). Symmetry of annealed wurtzite CdSe nanocrystals: assignment to the C3v point group. J. Phys. Chem. 100, 17417–17422. 10.1021/j100048a017

[B42] Shin Y. Lee J. Yang J. Park J. Lee K. Kim S.. (2014). Mass production of graphene quantum dots by one-pot synthesis directly from graphite in high yield. Small 10, 866–870. 10.1002/smll.201302286 24745051

[B43] Sudlow G. Birkett D. J. Wade D. N. (1976). Further characterization of specific drug binding sites on human serum albumin. Mol. Pharmacol. 12, 1052–1061. 1004490

[B44] Sun H. Yang X. Li M. (2015). Insights into the effect of N-acetyl- L -cysteine-capped CdTe quantum dots on the structure and activity of human serum albumin by spectroscopic techniques. J. Luminescenc 167, 1–7. 10.1016/j.jlumin.2015.06.005

[B45] Tabassum S. Al-Asbahy W. M. Afzal M. Arjmand F. Khan R. H. (2012). Interaction and photo-induced cleavage studies of a copper based chemotherapeutic drug with human serum albumin: spectroscopic and molecular docking study. Mol. Biosyst. 8, 2424–2433. 10.1039/c2mb25119a 22790833

[B46] Tang N. Zhou C. Xu L. Jiang Y. Qu H. Duan X. (2019). A fully integrated wireless flexible ammonia sensor fabricated by soft nano-lithography. ACS Sensors. 4, 726–732. 10.1021/acssensors.8b01690 30793588

[B47] Torimoto T. Adachi T. Okazaki K. Sakuraoka M. Shibayama T. Ohtani B.. (2007). Facile synthesis of ZnS– AgInS2 solid solution nanoparticles for a color-adjustable luminophore. J. Am. Chem. Soc. 129, 12388–12389. 10.1021/ja0750470 17887678

[B48] Venyaminov S. Y. Woody R. W. (1999). Estimation of the number of [alpha]-helical and [beta]-strand, segments in proteins using circular dichroism spectroscopy. Protein Sci. A Publi. Protein Soc. 8, 370–380. 10.1110/ps.8.2.370 PMC2144265 10048330

[B49] Wang X. Cui Y. Li T. Lei M Li J. Wei Z. (2019). Recent advances in the functional 2D photonic and optoelectronic devices. Adv. Optical Mater. 7:1801274 10.1002/adom.201801274

[B50] Zhang G. Que Q. Pan J. Guo J. (2008). Study of the interaction between icariin and human serum albumin by fluorescence spectroscopy. J. Mol. Struct. 881, 132–138. 10.1016/j.molstruc.2007.09.002

[B51] Zhou C. Zhang X. Tang N. Fang Y. Zhang H. Duan X. (2019). Rapid response flexible humidity sensor for respiration monitoring using nano-confined strategy. Nanotechnology 31:125302. 10.1088/1361-6528/ab5cda 31778983

[B52] Zu Z. Hu W. Tang X. Yang P. H. W. Chen W. Li S. (2016). A facile method for synthesizing AgInZnS/RGO nanocomposites and their photoelectric detection application. Mater. Lett. 182:240–243. 10.1016/j.matlet.2016.07.001

